# Supplementary material for: A Computational Model of Tumor Interactions with Bone-Resident Cells Predicts Tumor-Type-Specific Responses to Perturbations
Source: bioRxiv. 2026 Mar 19:2026.02.16.706164. Originally published 2026 Feb 18. Preprint. [Version 2] doi: 10.64898/2026.02.16.706164 (PMC12934782; doi:10.64898/2026.02.16.706164)
Supplement: 2 [file NIHPP2026.02.16.706164v2-supplement-2.pdf]

bioRxiv preprint doi: https://doi.org/10.64898/2026.02.16.706164; this version posted March 19, 2026. The copyright holder for this preprint (which was not certified by peer review) is the author/funder, who has granted bioRxiv a license to display the preprint in perpetuity. It is made available under aCC-BY 4.0 International license.

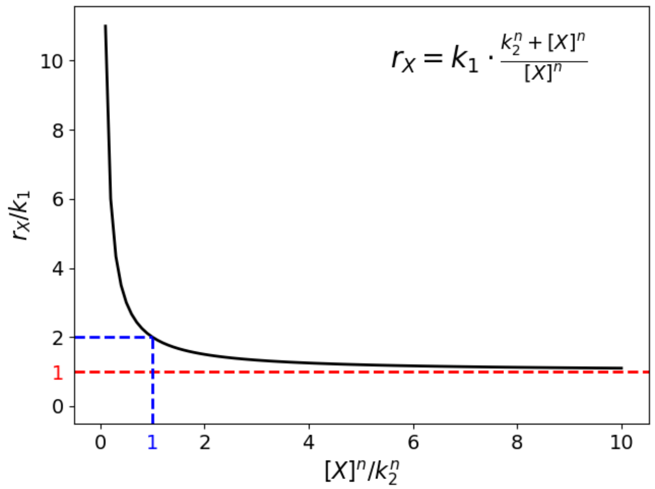

Figure S1. Illustration of the per-cell reaction rate expression used to model OB bone formation and OC bone consumption. The per-cell reaction rate, r_X, is shown in the top-right corner of the plot, while the axis values are scaled to non-dimensionalize the equation. The red dashed line indicates that at large values of $[X]$ (OBs or OCs), $r_X \rightarrow k_1$; the blue dashed line indicates that $r_X = 2k_1$ when $[X] = k_2$. Square brackets represent concentration.

bioRxiv preprint doi: https://doi.org/10.64898/2026.02.16.706164; this version posted March 19, 2026. The copyright holder for this preprint (which was not certified by peer review) is the author/funder, who has granted bioRxiv a license to display the preprint in perpetuity. It is made available under aCC-BY 4.0 International license.

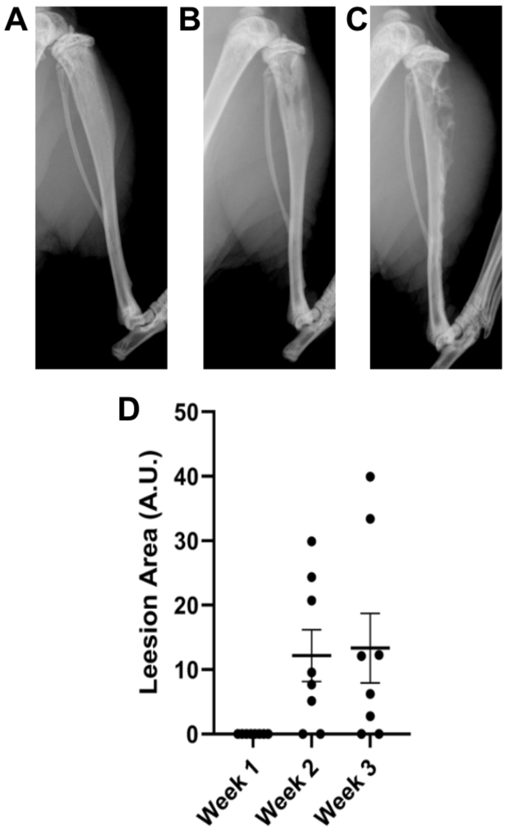

Figure S2. Representative x-ray images of mice after tumor injection. X-ray images show tibias of mice one week **(A)**, two weeks **(B)**, and three weeks **(C)** after MDA-MB-231b (bone-adapted) injection. **(D)** Quantification of lesion area (mean ± standard error). Mice with no lesions at two and three weeks after tumor cell injection were excluded from all downstream analyses. n = 8 per group.

bioRxiv preprint doi: https://doi.org/10.64898/2026.02.16.706164; this version posted March 19, 2026. The copyright holder for this preprint (which was not certified by peer review) is the author/funder, who has granted bioRxiv a license to display the preprint in perpetuity. It is made available under aCC-BY 4.0 International license.

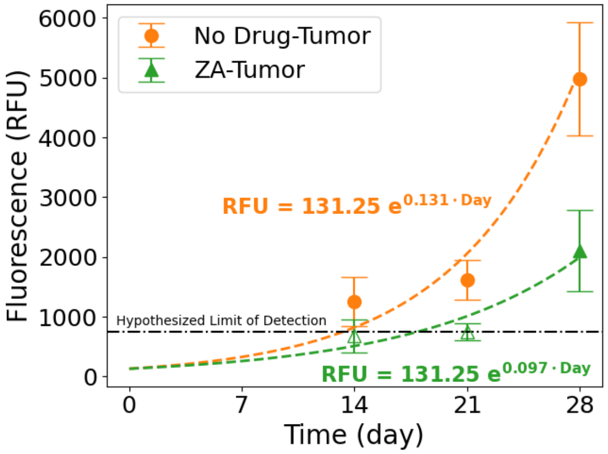

Figure S3. Exponential fits to fluorescence tumor burden data from Johnson et al. (16). Exponential prefactors for both datasets are purposely set equal since the number of injected tumor cells at Day 0 is the same in both cases. Also, data points for the ZA-treated tumor at 14 and 21 days post-injection (unfilled triangles) are excluded from the exponential fit since they fall below our hypothesized limit of detection (750 RFU). Assuming the injected tumor concentration is 1 fM at Day 0, these curves are used to estimate tumor cell counts at Day 7 for the untreated tumor ("No Drug–Tumor") and Days 7, 14, and 21 for the ZA-treated tumor ("ZA-Tumor"; *Materials and Methods*). RFU: relative fluorescence units; ZA: zoledronic acid.

bioRxiv preprint doi: https://doi.org/10.64898/2026.02.16.706164; this version posted March 19, 2026. The copyright holder for this preprint (which was not certified by peer review) is the author/funder, who has granted bioRxiv a license to display the preprint in perpetuity. It is made available under aCC-BY 4.0 International license.

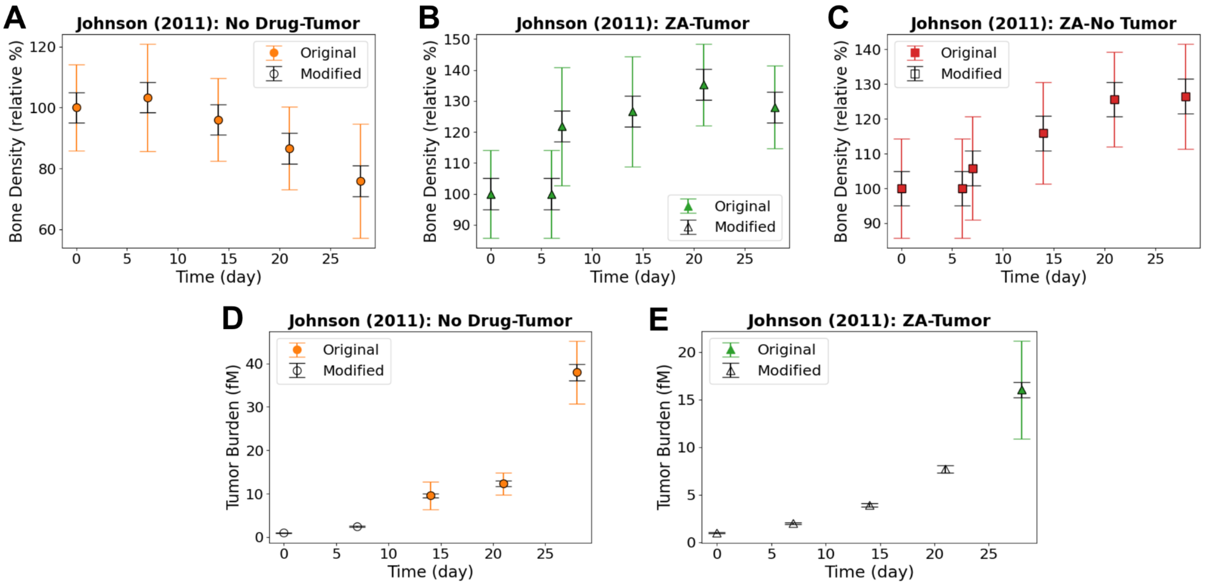

Figure S4. Comparison of original and modified error bars for the extracted datasets from Johnson et al. (16). (A–C) Bone density for untreated, tumor-bearing (*A*), drug-treated, tumor-bearing (*B*), and drug-treated, non-tumor-bearing (*C*) mice; **(D, E)** tumor burden for untreated, tumor-bearing (*D*) and drug-treated tumor-bearing (*E*) mice. Note that the "Modified" data points at Days 0 and 7 in panel *D* and Days 0, 7, 14, and 21 in panel *E* are estimated based on exponential fits to the original data points (see Fig. S3 and *Materials and Methods*). Thus, there are no "Original" data points for these cases.

bioRxiv preprint doi: https://doi.org/10.64898/2026.02.16.706164; this version posted March 19, 2026. The copyright holder for this preprint (which was not certified by peer review) is the author/funder, who has granted bioRxiv a license to display the preprint in perpetuity. It is made available under aCC-BY 4.0 International license.

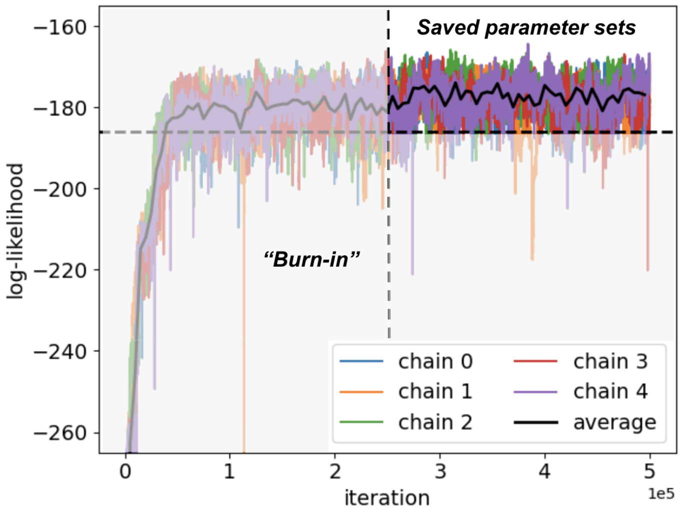

Figure S5. Log-likelihoods at each iteration for all five Markov-chain Monte Carlo (MCMC) chains during the PyDREAM calibration run. The run took a total of 5×10^5 iterations. The first 2.5×10^5 iterations were considered "burn-in" and discarded. The dashed horizontal line represents two standard deviations from the mean value of the log-likelihood, calculated over the last 2.5×10^5 iterations. Parameter sets with log-likelihood values below this line were discarded. In total, 1,212,105 parameter sets, of which 81,467 are unique, were retained after filtering.

bioRxiv preprint doi: https://doi.org/10.64898/2026.02.16.706164; this version posted March 19, 2026. The copyright holder for this preprint (which was not certified by peer review) is the author/funder, who has granted bioRxiv a license to display the preprint in perpetuity. It is made available under aCC-BY 4.0 International license.

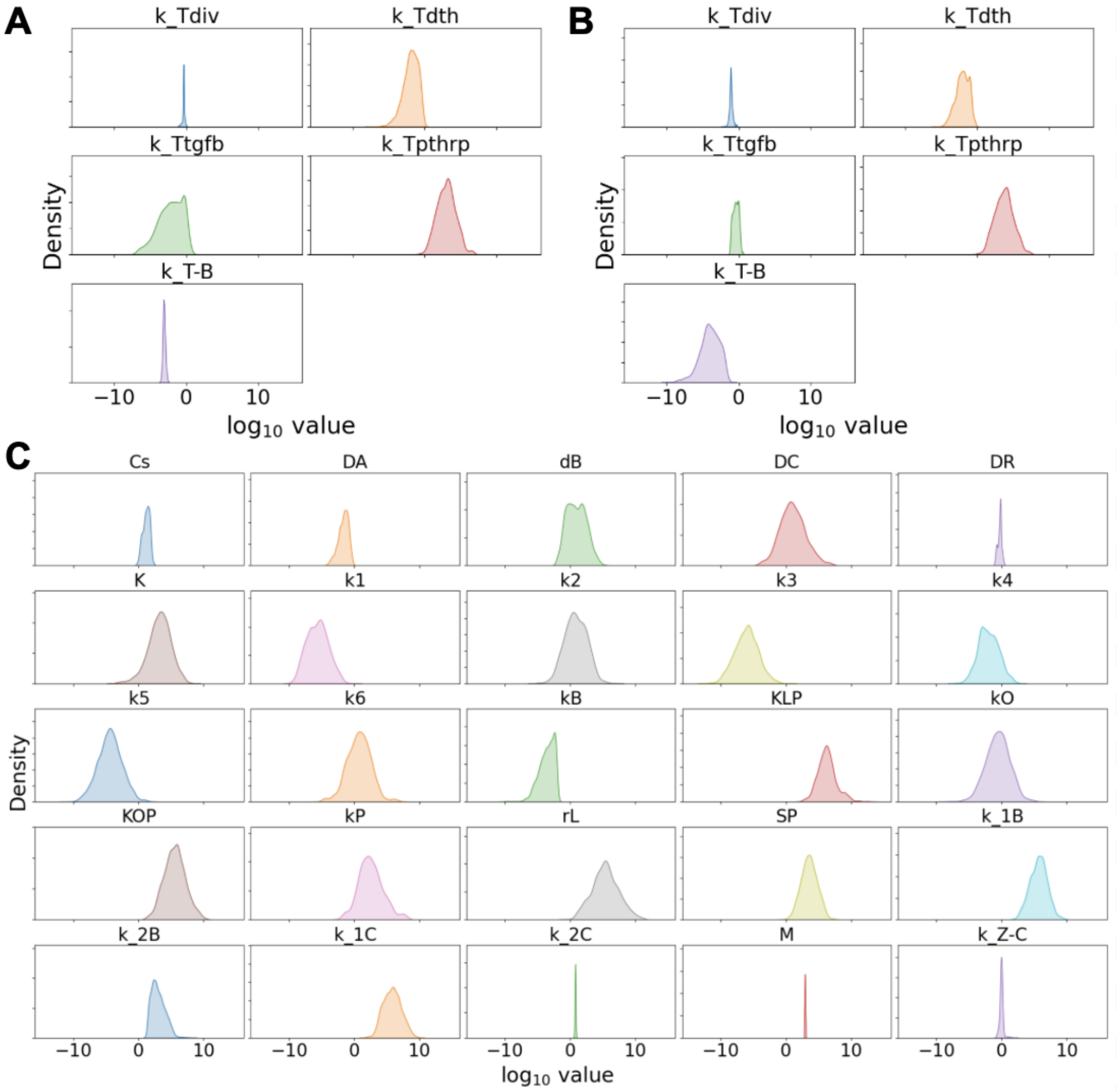

Figure S6. Distributions of parameter values inferred by PyDREAM. (A, B) The five tumor-associated model parameters for the *(A)* bone-adapted tumor (*this work*; see Figures 3, 5, and 6 of the main text) and *(B)* parental-derived non-adapted tumor (16) (see Figures 3 and 5 of the main text). Note that the distributions in *A* and *B* are overlaid in Figure 4A of the main text. **(C)** The 25 non-tumor-associated model parameters. All distributions are based on the 1,212,105 parameter sets sampled by PyDREAM. The x-axes are shared among all plots in *A*–*C*.

bioRxiv preprint doi: https://doi.org/10.64898/2026.02.16.706164; this version posted March 19, 2026. The copyright holder for this preprint (which was not certified by peer review) is the author/funder, who has granted bioRxiv a license to display the preprint in perpetuity. It is made available under aCC-BY 4.0 International license.

Table S1. Mechanistic model of OB, OC, tumor, and bone dynamics. Reactions 1-5 are from the Lemaire et al. bone homeostasis model (5). Reactions 6-12 were added in this work to capture interactions between tumor, bone, osteoblasts (OBs), and osteoclasts (OCs), as well as the effect of drug treatment with zoledronic acid (ZA). *R*: responding OB, *B*: active OB, *C*: active OC; *N*: bone density; *T*: tumor cell; *Z*: ZA. Square brackets indicate concentration.

Index	Reaction	Rate Expression	Description
		Bone-Resident Cells	
1	$\emptyset \rightarrow R$	$D_R \cdot \pi_C$	Responding OB production
2	$R \rightarrow B$	$f_0 d_B / \pi_C$	OB activation
3	$B \rightarrow \emptyset$	k_B	Active OB death
4	$\emptyset \rightarrow C$	$D_C \cdot \pi_L$	Active OC production
5	$C \rightarrow \emptyset$	$D_A \cdot \pi_C$	Active OC death
		Bone	
6	$B \rightarrow B + N$	$k_{1B} \cdot \frac{k_{2B}^{n_B} + [B]^{n_B}}{[B]^{n_B}}$	Active OBs build bone
7	$C + N \rightarrow C$	$k_{1C} \cdot \frac{k_{2C}^{n_C} + [C]^{n_C}}{[C]^{n_C}}$	Active OCs consume bone
		Tumor Cells	
8	$T \rightarrow 2T$	$k_{Tdiv} + k_{Ttgfb} \cdot \pi_C$	Cell division (basal + TGF-β-enhanced)
9	$T \rightarrow \emptyset$	k_{Tdth}	Natural cell death
10	$2T \rightarrow T$	$(k_{Tdiv} - k_{Tdth})/M$	Density-dependent cell death (carrying capacity)
11	$T + B \rightarrow T$	k_{T-B}	Tumor cells promote OB death
		Drug Treatment	
12	$Z + C \rightarrow Z$	k_{Z-C}	ZA promotes OC death

Table S2. Functions used in the rate expressions in Table S1. Functions 1-3 are from the Lemaire et al. bone homeostasis model (5). Function 4 was added in this work to capture PTHrP secretion from tumor cells. *R*: responding osteoblast (OB), *B*: active OB, *C*: active osteoclast (OC); *T*: tumor cell. Square brackets indicate concentration.

Index	Function	Units	Description
	Bone-Resident Cells		
1	$\pi_P = \frac{I_p/k_p + S_p/k_p}{I_p/k_p + k_6/k_5}$	unitless	Fraction of bound PTH receptors on ROBs and AOBs
2	$\pi_C = \frac{f_0 C^s + [C]}{C^s + [C]}$	unitless	Fraction of bound TGF-β receptors on uncommitted OB progenitors
3	$\pi_L = \frac{k_3}{k_4} \cdot \frac{K_L^P \cdot \pi_P \cdot [B]}{1 + \frac{k_3 K}{k_4} + \frac{k_1}{k_2 k_O} \cdot \left(\frac{K_O^P}{\pi_P}[R] + \bar{I}_O\right)} \cdot \left(1 + \frac{\bar{I}_L}{r_L}\right)$	unitless	Ratio of bound to unbound RANK receptors on OC precursors
	Tumor Cells		
4	$I_P = \bar{I}_P + k_{Tpthrp} \cdot \pi_C \cdot [T]$	$pM{\cdot}day^{-1}$	Rate of injected PTH/PTHrP, external and from tumor
